# Treatment related acute promyelocytic leukemia (t-APML) in breast cancer survivor treated with anthracycline based chemotherapy: rare case report

**Published:** 2015-10-01

**Authors:** Irappa Madabhavi, Gaurang Modi, Harsha Panchal, Apurva Patel, Asha Anand, Sonia Parikh

**Affiliations:** Department of Medical and Pediatric Oncology, GCRI, Ahmedabad, Gujarat, India

**Keywords:** Anthracycline, Breast cancer, t-APML

## Abstract

Treatment related acute myeloid leukemia (t-AML) is well documented phenomenon after chemotherapy. In this subgroup of patients acute promyelocytic leukemia (APML) due to delayed complication of using anthracycline is very rare occurrence. Very few cases are reported in world literature. We are reporting a rare case of occurrence of t-APML in cured breast cancer patient treated with doxorubicin. 43 year old female presented with triple negative early breast cancer treated initially with Right modified radical mastectomy. Pathological staging was pT2N0M0. She was treated with 6 cycle of adjuvant AC (Doxorubicin, Cyclophosphamide). After latent period of 23 months she developed symptoms of fever, weakness and generalized body ache. On further investigation she was found to have acute promyelocytic leukemia (APML). We had successfully treated t-APML with conventional 7+3 induction and subsequent consolidation with ATRA (All Trans Retinoic Acid) and arsenic trioxide. Patient was given maintenance treatment for 18 months after confirming negative PML RARA by RT PCR and declared cured. Patient is under regular surveillance in our centre.

## Introduction

 Anthracyclines are one the most common anticancer drugs used for haematological and solid malignancies. Therapy related APML (t-APML) after using anthracycline as adjuvant treatment in breast cancer is a rare entity. Very few cases are reported in the world literature till date. Treatment of t-AMPL and achieving cure is challenging. Even though anthracyclines are the causative agents for t-APML and using these drugs in the management of t-APML is an interesting feature.

## CASE REPORT

 43 year old pre menopausal female presented with right sided breast lump since 10 days before presenting in the clinic. Her past medical and family 

history was not significant. On examination there was a firm to hard lump in the right upper and outer quadrant of the breast of size 4 X 3 cm. The lump was freely movable and was not associated with any fixity to the skin or chest wall. Clinically, there was no axillary lymphadenopathy. Her preoperative baseline biochemical and radiological investigations were within the normal range. She was treated with right MRM (modified radical mastectomy) and pathological staging was pT2N0M0. Patient's cardiac function evaluated by 2D ECHO, which was normal. Patient was given 6 cycles of adjuvant AC. Dose of Doxorubicin was 60 mg/m2 and Cyclophosphamide was 600 mg/ m2 per cycle at the interval of 21 days. Total cumulative dose of Doxorubicin was 360 mg/m2.

Post operative radiation therapy was not given. She was under regular follow up at our cancer care centre with yearly normal mammosonography. After a latent period of 23 months patient developed fever, generalized weakness and body ache.

**Figure 1:**
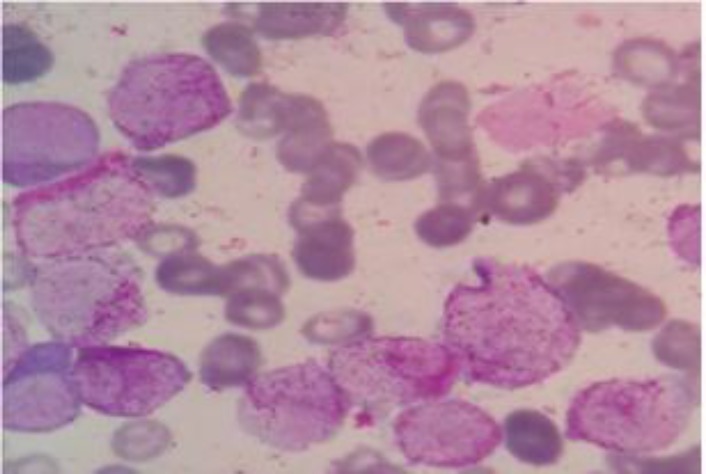
Bone marrow aspiration smear shows promyelocytes characterized by densely packed purple granules which obscure the nuclei and faggot cells which are packed with Auer rods. Morphologically, cells are hyper granular type and Auer rods were seen.

On examination her vitals were stable and her ECOG performance status was 1 (eastern co operative oncology group), height, weight and body mass index was within normal limit for her age. Purpuric spots were noted all over the body. Clinically, there was no lymphadenopathy and juandice. Rest of the systemic examination was within normal limit. Complete blood count shows HB of 5.7 gm%, white blood cells of 56000/cumm, and platelets counts of 12000/cmm. Her liver function test, renal function test, uric acid, lactate dehydrogenase, calcium and coagulation profile were normal. Peripheral smear shows 54% promyelocytes. Bone marrow aspiration smear shows 87% promyelocytes characterized by densely packed purple granules which obscure the nuclei and faggot cells which are packed with Auer rods. Morphologically cells are hyper granular type and Auer rods were seen (Figure 1). PML RARA translocation was positive by fluorescent in situ hybridization (FISH) method. 2D ECHO was done which showed normal ejection fraction. Conventional cytogenetic was normal.

Supportive treatment included packed cell volume, fresh frozen plasma and single donor platelet. ATRA was started at the dose of 45 mg/ m^2^/ day without any delay. After consent for treatment, she was given conventional 7+3 inductions (Cytarabine 200 mg/day for 7 days and Daunorubicin 60 mg/m^2^ for 3 days) along with ATRA. Patient tolerated induction therapy well and count recovery occurred at day 25 of induction. Repeat marrow was performed on the day 30 of induction which showed complete remission. ATRA was continued till 42 days of induction. Patient was consolidated with arsenic trioxide 0.15 mg/kg for 5 days a week for 4 weeks followed by 4 weeks of rest for 4 cycles and ATRA 45 mg/^2 ^per day for 2 weeks and 2 weeks of rest for seven cycles. PML RARA by reverse transcriptase polymerase chain reaction (RT PCR) was undetectable after consolidation therapy.

She was put on maintenance treatment inform of 6-mercaptopurine 50 mg daily and weekly oral methotrexate 15 mg/m^2^. ATRA was given at a dose of 45 mg/m^2 ^every 3 monthly for 15 days. Total duration of maintenance was 18 months. She was declared cured after again confirming molecular remission by RT PCR. Patient is under regular surveillance in our tertiary care centre with yearly mammosonography and monthly CBC and platelets since one and half year.

## Discussion

 Anthracyclines are one of the most common chemotherapy medications used worldwide. It is especially true for India as large number of breast cancer patients diagnosed early due to improved literacy rate and nationwide screening programs. True incidence of t-APML after using anthracycline is unknown due to rarity of its occurrence.^1^^,^^2^

Doxorubicin induced t-APML results from DNA cleavage by topoisomerase 2 and resulting genetic alteration in form of chromosome translocations and subsequent t-APML. Some evidence support a direct role of topoisomerase II in causing the DNA damage that leads to chromosomal rearrangements. An indirect mechanism involving induction of apoptosis inducing nucleases has also been proposed.^3^ Doxorubicin also causes an increase in the concentration of breakpoint complexes. All these mechanisms lead to cleavage of PML and RARA and formation of t (15; 17).

Pulsoni (51 patients), Beaumont (106 patients), Dayyani (29 patients) all these recent study are showing that breast cancer is the most common cancer in which t-APML occurred.^1^^,^^2^^,^^4^ Cumulative dose of anthracycline for secondary leukaemia is not defined. One study in pediatric patients favours dose more than 170 mg/m^2^ is considered leukemogenic.^5^

Median latent period of t-APML is 25-29 months. In our case, latent period was 23 months. Haematological and marrow characteristic of t-APML are same as primary APML but cytogenetic characteristics are different. There are more rearrangements in chromosomes 5, 7 or 17 and less trisomy of 8 are seen in t-APML as compared to non t-APML. In our case conventional cytogenetic was normal. Outcome is similar as in non t-APML.^2^

Rechallenging again with anthracycline as induction treatment of t-APML is an interesting aspect. Hu J et al. demonstrated Long-term efficacy and safety of 85 patients who diagnosed as de novo APML and received arsenic trioxide/ATRA.^6^ Chemotherapy was given as induction if white blood counts were more than 10,000, Chemotherapy was given in as induction if count white blood counts were more than 10,000, routinely in consolidation and in maintenance at a low dose. Complete remission (CR) was seen in 94.1% of patients. Five-year overall survival (OS) for all patients was 92%. Study concluded that prognosis is not influenced by day1 white blood cell counts, different subtypes of PML-RARA translocation, or FLT3 mutations

Dayyani. et al^4^ studied 29 patients of t-APML and compared outcome of who treated with ATRA + chemotherapy and ATRA + ATO. Author concluded later regimen did not detect inferior results compare to former. We did not use daunorubicin as consolidation treatment as cumulative dose for cardio toxicity that is 450 mg/m^2^ would be crossed (after calculating dose conversion with doxorubicin). Considering our limited resources, management of such case was challenging.

## CONCLUSION

 It is likely that parallel to increase of use of anthracycline for various malignancies, the incidence of t-APML has increased. Its presentation, management and outcome are similar to non t-APML Anthrayclines can be used during induction or consolidation therapy of APML therapy until the ceiling dose of the drugs are reached and later can be replaced with arsenic trioxide and it is clinically challenging and desirable**.**
